# Personalized *in silico* model for radiation-induced pulmonary fibrosis

**DOI:** 10.1098/rsif.2024.0525

**Published:** 2024-11-13

**Authors:** Eleftherios Ioannou, Myrianthi Hadjicharalambous, Anastasia Malai, Elisavet Papageorgiou, Antri Peraticou, Nicos Katodritis, Dimitrios Vomvas, Vasileios Vavourakis

**Affiliations:** ^1^ Department of Mechanical & Manufacturing Engineering, University of Cyprus, Nicosia, Cyprus; ^2^ Bank of Cyprus Oncology Centre, Nicosia, Cyprus; ^3^ Department of Medical Physics and Biomedical Engineering, University College London, London, UK

**Keywords:** radiation-induced pulmonary fibrosis, *in silico*, mathematical model, radiotherapy, lung cancer

## Abstract

Radiation-induced pulmonary fibrosis (RIPF) is a severe late-stage complication of radiotherapy (RT) to the chest area, typically used in lung cancer treatment. This condition is characterized by the gradual and irreversible replacement of healthy lung tissue with fibrous scar tissue, leading to decreased lung function, reduced oxygen exchange and critical respiratory deficiencies. Currently, predicting and managing lung fibrosis post-RT remains challenging, with limited preventive and treatment options. Accurate prediction of fibrosis onset and progression is therefore clinically crucial. We present a personalized *in silico* model for pulmonary fibrosis that encompasses tumour regression, fibrosis development and lung tissue remodelling post-radiation. Our continuum-based model was developed using data from 12 RT-treated lung cancer patients and integrates computed tomography (CT) and dosimetry data to simulate the spatio-temporal evolution of fibrosis. We demonstrate the ability of the *in silico* model to capture the extent of fibrosis in the entire cohort with a less than 1% deviation from clinical observations, in addition to providing quantitative metrics of spatial similarity. These findings underscore the potential of the model to improve treatment planning and risk assessment, paving the way for more personalized and effective management of RIPF.

## 1. Introduction

Lung cancer is one of the most common and serious types of cancer affecting both men and women worldwide, and it is most commonly diagnosed in elderly people [1]. These malignant tumours are characterized by uncontrolled cell growth in the lung and can spread beyond the organ into nearby tissue or other parts of the body. Focusing on primary lung cancer here, its treatment depends on its type, stage, size and location and the general health of the individual. The main treatments include surgery, chemotherapy, radiotherapy (RT), microwave or radiofrequency ablation or a combination of the above.

Radiation-induced pulmonary fibrosis (RIPF) is a common and severe complication of RT, affecting 5−50% of patients undergoing RT in the thorax [2]. It is identified as the latter stage of radiation-induced lung injury occurring 6–12 months after RT, while the early stage of lung injury from radiation is identified as pneumonitis and occurs in the first three to six months after RT. The development of pulmonary fibrosis is a complex dynamic process involving several interacting biological processes. Ionizing radiation induces direct damage to the DNA of the cells and indirect damage through the production of reactive oxygen/nitrogen species. This leads to a cascade of patho-physiological processes that is not yet completely understood [2–4]. It starts with an immune system response to cell damage which leads to inflammation. The initial pro-inflammatory response is followed by a wound-healing response which involves the recruitment of fibroblasts and their transdifferentiation into myofibroblasts. This leads to collagen deposition and remodelling of the extracellular matrix (ECM) of the lung. If this process is prolonged or dysfunctional, it leads to excess collagen deposition which causes tissue thickening and stiffening, resulting in pulmonary fibrosis.

RIPF reduces lung tissue compliance and, therefore, physiological lung functionality [5]. It disrupts multiple aspects of the physiology of the lung, including efficient gas exchange, thus, adequate airflow and the optimal balance between lungs’ ventilation and tissue perfusion [6]. Several risk factors have been identified for RIPF such as total radiation dose, fraction size, lung volume treated and adjuvant chemotherapy, in addition to patient-related variables (e.g. age, comorbidities and genetics) [3,6]. Furthermore, RIPF shares several commonalities with the pathways that promote irreversible damage to the epithelium of lung airways and the degradation of the lung parenchyma [7]—as with the two other major respiratory diseases: chronic obstructive pulmonary disease and idiopathic pulmonary fibrosis (IPF). Several recent studies have been focused on identifying molecular and genetic biomarkers associated with clinical outcomes of fibrosis in the lung [8,9] and have employed *in vitro* fibrosis models and *in vivo* animal models to study the pathology of the disease [10,11].

Despite advances in laboratory tests [4,12], there are still many challenges to elucidate the pathophysiology of pulmonary fibrosis and develop personalized therapeutic strategies to combat the disease [2]. Computational models, referred to as *in silico* models, and medical image computing may help clinicians identify which patients are more likely to develop RIPF and therefore support radiologists to tailor lung cancer management on a patient-specific basis [13,14]. *In silico* modelling is gaining significant momentum and has improved substantially in producing realistic and physiologically relevant simulations of lung biomechanics [15]. The review of Leonard-Duke *et al.* [16] presents a comprehensive summary of *in silico* models on the biology and biomechanics of pulmonary fibrosis, with focus on idiopathic and tuberculosis-related pulmonary fibrosis. In the following, we provide a brief overview of the most notable works related to lung fibrosis modelling.

Computational models used to simulate pulmonary fibrosis can be broadly classified into two categories based on how they represent the biological components of the system: discrete models and continuum models. Discrete models represent individual biological components (such as cells, molecules or organisms) as distinct, separate entities. In the work of Bates *et al.* [17,18], they proposed a two-dimensional network model of linear elastic springs to simulate the biomechanics of lung tissue in pulmonary fibrosis and emphysema. They then modelled fibrosis by randomly stiffening individual springs, while in emphysema, springs were pruned from the network as they failed under some critical tension. This contribution illustrated that mechanical dysfunction (that accompanies pulmonary fibrosis and emphysema) follows a very different course from the progression of the underlying microscopic pathophysiology itself, particularly in the early stages of disease. Continuum models, on the other hand, represent biological components using generalized descriptions that assume homogeneity within the system. Ordinary differential equations (ODEs) and/or partial differential equations (PDEs) are commonly employed in this class of models to describe the temporal and spatial dynamics of biological processes, respectively. Liu & Yang [19] used ODEs to model the dynamics and interactions between healthy and cancerous cells under constant radiation delivery. The case of periodic radiation delivery has been performed in a previous study by Liu *et al.* [20], where they presented a mathematical model to study the growth circumstances of the cell populations. They also established with their model the conditions on the permanence and regression of the normal and radiated cells.

Many recent studies combine different modelling techniques to create hybrid models. Wellman *et al.* [21] developed a hybrid *in silico* model that couples an agent-based model and a mechanistic model to simulate peripheral honeycombing in the lung, driven by stroma tissue patterns observed in histology [22]. They simulated several key aspects of the development and progression of idiopathic lung fibrosis, such as fibroblasts activation and movement and tissue stiffening. The model was successful in replicating the spatial progression of fibrosis, particularly how it starts in the sub-pleural regions of the lung and spreads inward. However, the simulation domain was two-dimensional and the clinical data used for comparison included only three patients.

Computational models of lung fibrosis can also be classified based on the biological scales they span, ranging from the molecular level to the cellular, tissue and whole-organ levels. Hao *et al.* [23] proposed a mathematical model for IPF represented by a set of PDEs that describe the dynamics of relevant cell populations. It operates at the cellular level and involves interactions between fibroblasts, myofibroblasts, macrophages and monocytes as well as proteins that regulate the progression of the disease, i.e. transforming growth factor beta, platelet-derived growth factor, matrix degrading enzymes, etc. Their model successfully simulated the progression of idiopathic fibrotic damage in the lung over time and was subsequently used to test the outcomes of two anti-fibrotic drugs for the treatment of IPF. However, their simulations focused on a small, cubic section of lung tissue and lacked direct validation with clinical data from human patients.

Rutkowska *et al.* [24,25] presented a whole-lung continuum model to study RT-induced damage to lung tissue, particularly focusing on the development of radiation pneumonitis. The model identifies a critical dose threshold for radiation-induced lung damage and demonstrates how different doses and volume effects influence the likelihood of developing radiation pneumonitis. However, they did not consider lung fibrosis and the organ was represented by a three-dimensional array of voxels, without considering the actual geometry of the lung.

More recently, Cogno *et al.* [26] developed an agent-based model of a single alveolar duct to simulate cell dynamics (growth, mitosis, senescent clearance, intra/inter-alveolar bystander senescence) and the diffusion of biochemical cues following irradiation. They followed a mechanistic approach which accounts for several biological processes related to radiation damage to epithelial cells and fibrosis development due to ECM accumulation. They replicated early and late patterns of pulmonary fibrosis growth as reported in the literature and showed qualitative agreement of a disease severity measure with published fibrosis indices, whereas their alveoli survival predictions diverged from the traditional linear-quadratic (LQ) model. The *in silico* approach of Cogno *et al.* [26], as with the hybrid model of Wellman *et al.* [21], are able to resolve spatial scales that extend up to the alveolus (micrometre) level, but the extension of the model to the lung organ scale poses a significant computational challenge for these models. While such models can provide detailed insights into cellular dynamics, they lack the scalability and integration with clinical imaging data needed for patient-specific, organ-wide applications, thus limiting their practical usability in a clinical setting.

In this contribution, we present a computational procedure for a personalized, whole organ *in silico* model of RIPF in lung cancer patients treated with external beam radiation. Our *in silico* model is continuum-based and can simulate the spatio-temporal progression of radiation-induced fibrosis as well as tumour regression and tissue remodelling. A key novelty of our model is its ability to simulate fibrosis at the whole-organ level, overcoming the scaling challenges faced by more localized models. Furthermore, it is personalized, incorporating patient-specific information such as the lung geometry, radiation dosage map distribution data, tumour features (location, size, shape) and lung tissue structural characteristics derived from the clinical CT scans. The model is calibrated and assessed against new clinical data from a cohort of 12 lung cancer patients, including delineations of fibrotic regions at two time points. The low computational cost and ability to integrate patient-specific clinical data offer a strong potential for clinical translation and real-time application in treatment planning and monitoring.

## 2. Methods

### 2.1. Lung cancer patient cohort

The data for this study are obtained from a retrospective cohort of cancer patients diagnosed with RIPF as a result of external beam radiation treatment for primary lung tumours. The cohort encompasses eight males and four females with ages ranging from 54 to 72 years old. Patients were diagnosed with various types of primary tumours in the lung that include lung adenocarcinoma (LUAD), lung squamous cell carcinoma (LUSC), lung adenosquamous carcinoma (LUASC), previously categorized into one histological subtype known as non-small cell lung cancer (NSCLC) [27], as well as one patient with small cell lung carcinoma (SCLC). The patients were treated with a combination of radiation and chemotherapy. Two radiation therapy techniques were used with eight patients treated with the conventional three-dimensional conformal RT technique, while four patients were treated using volumetric modulated arc therapy, which is capable of more precise shaping of the radiation distribution to the tumour. Chemotherapy consisted of platinum doublet regimens for all patients. Additionally, two patients received immunotherapy. Complete clinical information for all patients is presented in electronic supplementary material, S1.

**Figure 1.**
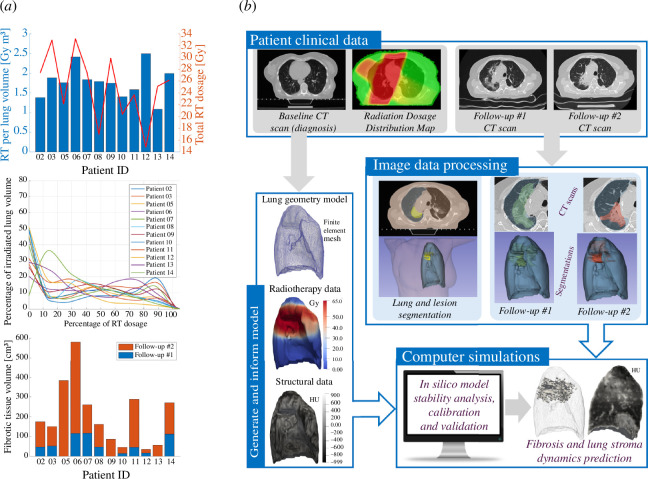
(*a*) (Top to bottom) Bar plot of the specific total dosage overlaid with the total RT dosage delivered (in Gy), the planned target volume expressed as a percentage of the lung volume that received irradiation as computed from the CT scans and bar plots of the clinically reported fibrotic tissue volume for all lung cancer patients. (*b*) Outline of the methodology used in the study which includes data processing and *in silico* simulations for calibrating and assessing the model predictions. The baseline CT images are used to produce segmentations of the lung and tumour which are then used to construct the mesh and extract initial values of the model variables. Subsequently, these are used by the computational solver to perform patient-specific simulations. An optimizer is used to calibrate the model parameters by comparing with the extent of fibrosis observed in the follow-up CT scans which have been processed to extract the segmentations of the fibrotic region. Finally, the predicted fibrosis is further compared with the actual fibrosis segmentations using image similarity indices.

The dataset consists of CT images prior to treatment (baseline scans) along with RT dosage plans that provide the radiation dose distribution within the lung. Additional CT images were provided at two time points after treatment (follow-up scans); the first was between two and six months and the second between 9 and 18 months from the date of start of radiation treatment. The treatment involved two RT plans: a broad RT plan covering a larger area around the tumour, administered daily in 28 fractions and a focused plan restricted specifically to the tumour region, administered daily in eight fractions. Both plans are integral parts of the therapy and deliver an equivalent radiation dose. Figure 1*a* depicts the RT data for all cancer patients.

Patient data were obtained from the Bank of Cyprus Oncology Centre (BoCOC), selected by treating physicians with the main inclusion criterion being clear development of fibrosis without additional health conditions beyond cancer and lung fibrosis. Patients were not subjected to any additional procedures or examinations for the purposes of this study. An expert radiologist at BoCOC labelled the fibrotic regions in these follow-up scans, providing crucial data for model validation. The clinical data were collected, anonymized and coded at BoCOC before being used for image processing, which is necessary for the model set-up and the simulations. Our study was approved by the National Bioethics Committee in Cyprus (case file: EEBK_EΠ_2021.01.83).

### 2.2. Medical image data processing

The patient data are processed to produce the patient-specific files required for the simulations (finite element mesh, initial data and radiation data), while segmentations of the fibrotic regions are used for calibrating and assessing the *in silico* model accuracy and fidelity. The core steps of the data processing pipeline are illustrated in figure 1*b*, which also presents the dataset used for each patient case, i.e. the CT images and the RT dosage maps. The baseline CT is used to produce a segmentation of the lung which is then used to create the computational mesh. Only the side of the lung that was irradiated is employed and therefore further processed. Subsequently, data from the medical images were used to assign initial values to model variables (i.e. tumour and lung tissue radiodensity) on each of the finite element mesh nodes (see electronic supplementary material, figure S3). These are then used by the computational software to perform the RIPF simulations and the results are compared with segmentations of the fibrosis extracted from the two follow-up scans. Image registration was performed between the follow-up scans and the baseline scan. This is required to allow for a direct comparison between the *in silico* results and the clinically measured regions of fibrosis. The exact details of the mesh generation, setting of initial values and registration procedure are provided in electronic supplementary material, S2.

### 2.3. Mathematical model of radiation-induced pulmonary fibrosis

The mathematical model considers a continuum-based formulation to capture the interactions and dynamics of lung tissue response to radiation treatment with a simplified approach, focusing on the most basic aspects of pulmonary fibrosis. It consists of two reaction–diffusion equations that govern the evolution of lung cancer cells and fibroblasts, while the remodelling of the lung tissue radiodensity [28] is modelled using an ODE. All state variables are directly affected by RT: tumour cells are reduced due to the cytotoxic effects of ionizing radiation, fibroblasts are recruited due to lung injury caused by radiation, and excessive lung tissue stroma is produced by the increased fibroblast population. The simplicity of the mathematical approach is driven by the fact that the precise mechanisms promoting the development of fibrosis are not completely understood [2–4,29]. Moreover, encompassing all currently known processes that influence pulmonary fibrosis would lead to a complicated model with numerous model parameters that are challenging to determine, as well as considerably increasing the computational burden. Instead, the use of a moderately simple mathematical model provides more tractable equations that can be easily analysed as well as a small number of model parameters that can be efficiently calibrated using pertinent clinical patient data.

The model consists of a set of coupled differential equations that describe the spatial and temporal evolution of the tumour cells in the lung (c, expressed as normalized cell density of lung cancer cells), the fibroblasts (f, normalized cell density of fibroblasts) and the lung tissue radiodensity (ϵ, expressed in Hounsfield units (HU)). Thus, c,f∈[0,1] represent normalized cell density and are dimensionless state variables, while ϵ is obtained directly from the CT images (expressed in the HU scale). The tumour cells population is governed by the PDE


(2.1) $$\dot{c}(x;t)=\underset{\text{migration}}{\underbrace{\nabla \;\cdot [D_{c}\;T\;\nabla c]}}-\underset{\text{apoptosis}}{\underbrace{\theta_{c}\;c\;Q}}+\underset{\text{proliferation}}{\underbrace{\lambda_{c}\;T\;(1\;-\;c)\;c}}\;,$$

where the dot notation expresses a time derivative. The first term represents cancer cell invasiveness modelled as isotropic diffusion, the second term represents cell death due to radiation and the third term represents tumour growth and relapse. This reaction–diffusion equation is adapted based on the proliferation–invasion (PI) model of Swanson *et al.* [30] for neoplastic cells’ migration and growth. Cell migration is generally directional and guided by ECM cues, but is often represented as an isotropic process in biological models as a simplifying assumption [31]. The LQ model for cell death due to radiation is a well-established one in the mathematical oncology community [32]. Function Q models cell death as a function of the radiation dose, R𝖣, in gray (Gy) units, through the formula: $Q(𝖣)=1-e^{-\alpha 𝖣-\beta {𝖣}^{2}}$, with parameter α determined by patient data and β determined based on the fixed ratio α/β=10, as in [33,34]. Apoptosis of cancer cells due to inadequate oxygenation or acidosis was not included since cancer regression is assumed to be caused predominantly by RT. Finally, the diffusion and production terms are restricted by the capacity function that reads: $T(c,f,\epsilon )=1-[c+f+0.5(1+\epsilon /\tilde{\epsilon }){]}^{\tau }$, where τ is a positive scalar parameter. As state variable ϵ takes values within the HU scale, it is included in a term that is scaled and adjusted to be in the range (0,1) in accordance with other state variables and $\tilde{\epsilon }=1000$ is the corresponding reference value. The capacity function T is dimensionless, with range (0,1], and provides a restriction to the movement and growth of cells in highly dense lung tissue. This function ensures that the combined presence of these components does not exceed the physiological capacity of the lung tissue density.

The second PDE governs the dynamics of fibroblasts


(2.2) $$\begin{array}{r} \begin{array}{rl} \dot{f}(x;t) & =\underset{\text{migration}}{\underbrace{\nabla \;\cdot [D_{f}\;T\;\nabla f-H_{f}\;T\;f\;\hat{\nabla }\epsilon ]}}-\underset{\text{death}}{\underbrace{\theta_{f}\;f}}\\ & +\underset{\text{recruitment}}{\underbrace{\kappa_{f}\;T\;(1\;-\;f{)}^{\mu }\;\frac{D(x)}{\tilde{D}}}}+\underset{\text{proliferation}}{\underbrace{\lambda_{f}\;T\;(1\;-\;f)\;f^{\nu }\;\frac{D(x)}{\tilde{D}}}}\;, \end{array} \end{array}$$

where the first two terms represent fibroblasts’ migration: $D_{f}$ is the isotropic diffusion rate coefficient, while cell haptotaxis (expressed through parameter $H_{f}$) is dependent on the gradient of ϵ to drive RIPF spread in the direction of low lung tissue densities. Fibroblast migration is generally influenced by the ECM microstructure and can have preferential directions. However, to account for all potential behaviours, we consider both isotropic (diffusion) and anisotropic (haptotaxis) migration processes. The weight of these two mechanisms is represented by their respective coefficients, which are determined through the optimization procedure. The following mathematical terms represent natural cell death and the recruitment and proliferation of fibroblasts which depend on the radiation dose, 𝖣, delivered to the lung. Initially, the fibroblasts cell density is set to zero, and the recruitment term governs the production of the first fibroblasts according to the radiation dose, while the proliferation term governs the late stages of production.

The radiation dosage follows the specific RT protocol that was administered to patients. It utilizes two RT plans applied in a series of therapeutic sessions. The first plan has a broad radiation field and is applied in 28 daily fractions, whereas the second plan has a focused distribution which is closer to the tumour region and is applied in eight daily fractions. Thus, the radiation dose, 𝖣, increases daily using the broad radiation field for the first 28 days and the focused radiation plan for the following 8 days. The third equation of the RIPF model describes the radiodensity of lung tissue that is represented by state variable ϵ. This variable take values from the corresponding Hounsfield scale [28], as in the data from the CT images themselves. The evolution of lung tissue is therefore determined by the changes in the tumour and fibrosis, and is defined by the ODE,


(2.3) $$\dot{\epsilon }(x;t)=\left(1+\frac{\epsilon }{\tilde{\epsilon }}\right)\left(\phi_{c}\;\dot{c}(x;t)+\phi_{f}\;\dot{f}(x;t)\right),$$

where scalar parameters $\phi_{c}$, $\phi_{f}$ regulate the effect of tumour shrinkage and fibroblasts growth on the observed HU signal respectively. The multiplier term ‘$1+\epsilon /\tilde{\epsilon }$’ describes a linear dependence of the change in radiodensity to the local value of ϵ. This provides heterogeneity in the growth/reduction rate and avoids uniform change of the ϵ field. In this study, lung tissue remodelling is dominated by tumour cells regression (due to RT) and the fibroblasts production that translate into changes in the HU scale, as it is clinically reported in CTs.

The development of the *in silico* model of RIPF involves discretizing all aforementioned equations and solving them numerically using a finite element solver. Specifically, the equations are formulated as a nonlinear system and solved with an implicit solver. The equations are advanced in time with a prescribed time step that is determined by a convergence test for each patient case, to ensure that it does not influence the solution. Moreover, the model calibration is performed using a variational Bayesian Monte Carlo optimization approach, while the assessment of the simulator is performed using established image similarity indices: we employed the DICE and the RAND index. Detailed technical information about the finite element discretization, the model implementation, calibration and assessment are provided in electronic supplementary material, S3.

## 3. Results

We present the numerical findings of the *in silico* model in simulating the dynamics of lung cancer cells, fibroblasts and lung tissue radiodensity in RIPF. Simulations were performed for all 12 patients of the cohort using the acquired clinical data, with the results organized into three main parts: first, we present the results of tumour regression after radiation treatment. Next, we explore the dynamics of fibrosis development, i.e. the spread of the disease in the lung and the disease progression trajectory, and then we compare the calibrated model against clinical evidence. Finally, we present our findings on lung tissue thickening and compare the *in silico* output against CT images from patients.

### 3.1. Simulating lung tumour regression following radiation treatment

In this paragraph, the results of the *in silico* model to simulate tumour shrinkage after RT are presented. The post-treatment monitoring protocol considered in this cohort of patients included only two follow-up scans, with the earliest happening in three to six months after treatment. This led to having no detailed information available on the evolution of the tumour from the follow-up CTs. Therefore, the parameters for equation (2.1) are obtained from the literature and adjusted to ensure tumour regression within a typical four weeks period [35,36] which corresponds to early responding tumours.

Parameters are assumed to be uniform across all patients, despite expected variations due to the different lung cancer types and patient-specific factors—this is outside the scope of the study and is left for future work. The *in silico* model combines the PI model with the LQ model for radiation-induced cell death. The PI model has two parameters: the coefficients for diffusion $D_{c}$ and proliferation $\lambda_{c}$, which we derive from related work in the literature. Jackson *et al*. [37] suggest $\lambda_{c}=0.058\;d^{-1}$ and $D_{c}=0.038$–$0.233\;mm^{2}\;d^{-1}$, whereas Rockne *et al*. [32] propose $\lambda_{c}=0.003$–$0.088\;d^{-1}$ and $D_{c}=0.016$–$0.888\;mm^{2}\;d^{-1}$ using patient data from 29 tumours. These studies concern primary brain cancers, where the PI model has been extensively applied to and are not necessarily applicable to lung cancer types. However, in the absence of relevant evidence to set our model parameters for equation (2.1), we adopt $\lambda_{c}=0.05\;d^{-1}$ and $D_{c}=0.1\;mm^{2}\;d^{-1}$ such that they are within the aforementioned ranges, while they have been also able to give realistic tumour dynamics simulation outputs.

In contrast, the LQ model has been extensively used for lung cancer RT modelling, and studies suggest values for the ratio α/β in the range of 8–16 Gy [38,39]. However, the value α/β= is well established and applicable to lung cancers [40] and thus adopted in this study. The second parameter of the LQ model, α=0.003 is selected such that the probability of cell death is monotonically increasing throughout the range 0–65 Gy, which is the range of the applied radiation for the current patient cohort.

**Figure 2.**
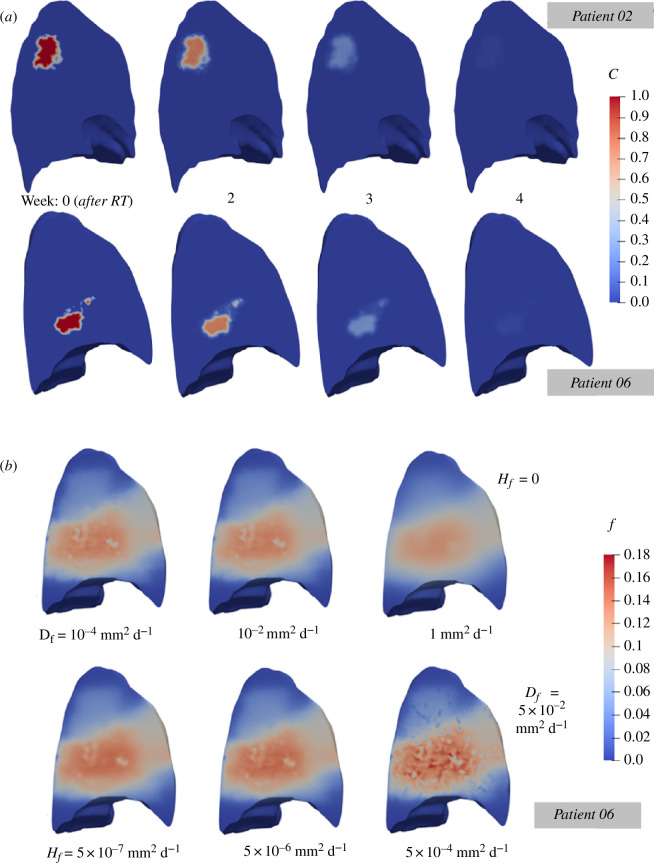
Cut-through of the Patient 02 and Patient 06 finite element three-dimensional model to visualize contour plots of simulation results. (*a*) Contours of state variable c (normalized tumour cell density) that illustrate the *in silico-*predicted tumour regression at weekly intervals. (*b*) Contours of state variable f (normalized fibroblasts density) showing the impact of varying diffusion (top row) and haptotaxis (bottom row) rate on fibrosis spread. Higher diffusion coefficients result in a smoother, more homogeneous fibrotic region, while higher haptotaxis coefficients create a more heterogeneous structure, i.e. honeycombing, that better reflects pulmonary fibrosis.

Figure 2*a* depicts the simulated lung cancer regression at weekly intervals for two representative cases, Patients 02 and 06 are indicative of the general behaviour observed across the entire patient cohort. The colour-coded maps represent the normalized cell density of the tumour cells, with hot intensity indicating the tumour enhancing region. The simulation starts with the lesion at maximum tumour cell density which shrinks by week 2—note the reduction in the tumour cell density in figure 2*a* (second and third column). From week 3 and onward, considerable regression of the carcinoma is evident, with only small clusters of tumour cells remaining that are diminished by week 4. This indicated complete tumour eradication by the end of the fourth week, in line with the clinical reports of the present study. The simulation results also demonstrate that the tumour dynamics are dominated by the LQ model, leading to significant reduction over the treatment period, and represent a successful RT treatment. Similar patterns of cancer regression are observed across the entire patient cohort on this study.

In conclusion, the lung carcinoma regression results underscore the potential of our computational model to realistically simulate the temporal evolution of tumour cells in response to RT. The results are indicative of general behaviour and do not represent a fully accurate trajectory of cancer treatment as this is not the primary focus of this work. However, the inclusion of tumour dynamics is crucial in order to capture the interplay between tumour, fibroblasts and lung tissue density and allow for a realistic representation of tissue evolution in our model.

### 3.2. Model recapitulates fibrosis development months after radiation treatment

This section presents the *in silico* results on the evolution of the fibroblasts cell population, its dependence on transport processes and the ability of the model to capture the behaviour observed in patient data. The analysis of the *in silico* RIPF model is divided into two parts. The first part focuses on the transport processes that model fibrosis spread, specifically the mathematical terms of diffusion and haptotaxis for fibroblasts’ migration. In the second part, we present patient-calibrated results and compare the simulated evolution of fibroblasts against the ground truth fibrosis measured from the patient data (follow-up CT scans). We then provide quantitative comparisons for the total volume and spatial similarity of the fibrotic region between the simulations and the clinical evidence.

#### 3.2.1. Diffusion and haptotaxis accurately reproduce pulmonary fibrosis spread

We explore the impact of the transport mechanisms, specifically diffusion and haptotaxis, on the spatial distribution and formation of RIPF. These mechanisms are critical in determining the overall structure and progression of the disease in the lung cancer patients after RT.

The effect of varying the diffusion coefficient on fibroblast distribution is presented in figure 2*b*, where the simulated fibrosis formation for different representative values of the diffusion coefficient, with the haptotaxis mechanism being deactivated, is illustrated. As the diffusion rate increases, we observe that the fibrotic region becomes more homogenized and smooth. High diffusion values result in a loss of detailed structure, transforming the fibrotic area into a homogeneous cluster. This smoothing effect contrasts sharply with the honeycomb-like structure of fibrosis seen clinically [22], indicating that diffusion should be limited to preserve the intricate architecture of scarring in the organ. Thus, an intermediate value of 5 × 10^−2^ mm^2^ d^−1^ is used as the initial value of the diffusion coefficient.

The impact of varying the haptotaxis coefficient is shown in figure 2*b*, where the diffusion coefficient, $D_{f}$, is kept constant at 5 × 10^−2^ mm^2^ d^−1^. In contrast to diffusion, increasing the haptotaxis coefficient results in more pronounced heterogeneity within the fibrotic region. Higher haptotaxis values lead to the formation of more extreme lows and highs in fibroblast-normalized cell density, f, hence producing more inhomogeneous structure that better resembles the actual fibrotic tissue seen in the radiological data. However, excessively high haptotaxis values can lead to unrealistic gaps with no apparent fibrosis inside the main fibrotic region and also pose numerical stability challenges. The value of 5 × 10^−5^ mm^2^ d^−1^ was selected as the initial value for the haptotaxis parameter, $H_{f}$.

The simulation results in this paragraph reveal the importance of carefully balancing the respective processes of fibrosis movement (in the mathematical model) to achieve pathophysiologically relevant structures of RIPF. The values determined from this investigation function as initial values for the optimization procedure that follows, which will calibrate the *in silico* model parameters using patient data.

#### 3.2.2. Radiation-induced pulmonary fibrosis model calibration to patient data

The simulation results presented in this paragraph focus on the evolution of fibrosis within the lung post-RT. To investigate the model’s ability to capture individual patient variability, the variational Bayesian Monte Carlo optimizer was employed to determine individualized model parameters—details of this procedure are in electronic supplementary material, S3.2. During the calibration process, it was found that the model’s parameters can be reduced without sacrificing the ability of the model to capture all of the studied cases. Specifically, it was observed that the exponent parameters can be fixed to μ=2 and ν=1 such that the variability introduced by the other five parameters of equation (2.2) was sufficient to produce evolution of fibrosis that matched each of the patients’ follow-ups. The values for the parameters of the fibroblast PDE are given in electronic supplementary material, table S3. Subsequent analysis delves into three representative cases, each characterized by a distinct magnitude of fibrosis volume: small size (Patient 12), medium size (Patient 02) and large size (Patient 06) of RIPF.

**Figure 3.**
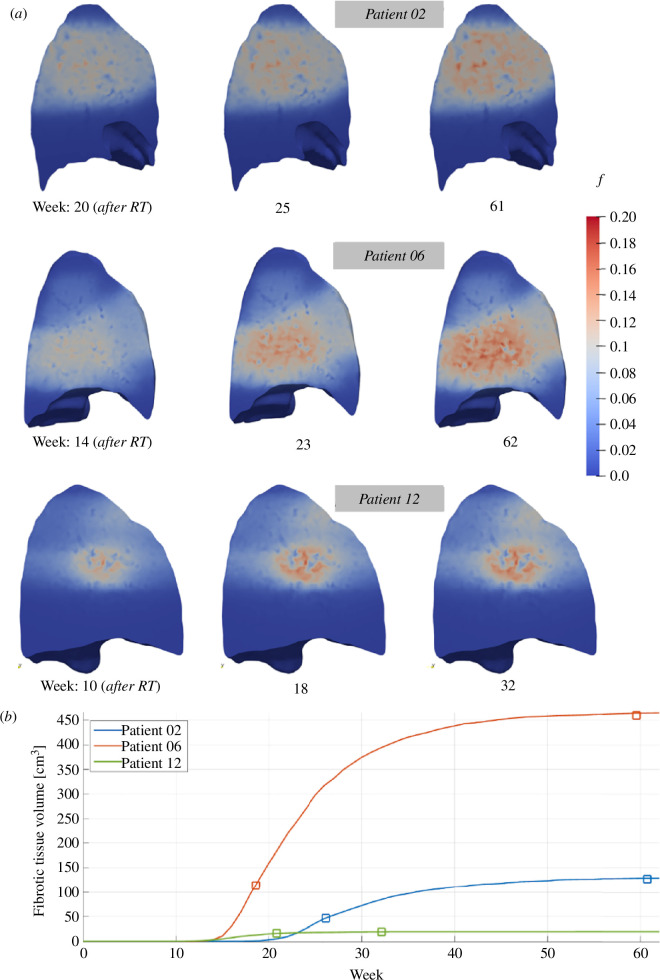
(*a*) Fibrosis progression for Patients 02, 06 and 12 that illustrate the development of medium, large and small extent of fibrosis in the lung after RT. (*b*) Time plots of the simulated volume of the fibrotic region for the same patients are compared against the clinically measured RIPF on the follow-ups for each patient, marked as open squares. This showcases the ability of the *in silico* model to capture the significantly diverse range of fibrosis development as illustrated by these example cases.

Figure 3*a* shows the *in silico*-predicted fibrotic region for each patient case at selected time points that correspond to just after initial development, at an intermediate time point and at the final time point where fibrosis has fully developed. In all patients, the regions of developing fibrosis follow the patterns of the RT plan and are seen to portray the beams intersecting around the target of the radiation treatment. However, the degree of fibrosis developed in regions away from the RT target is only minimal and has insignificant impact on tissue regeneration.

To quantify the evolution of fibrosis, line plots illustrating the volume of the fibrotic region over time are presented in figure 3*b*. The plots include data points representing the actual fibrosis volume measured at two follow-up time points. There is excellent agreement between the calibrated model results and the clinically measured fibrosis volumes, underscoring the model’s ability to capture the dynamics of RIPF progression for all three cases, which vary significantly in terms of the extent of developed fibrosis.

The *in silico* model is further tested by quantitatively comparing the simulated results against the patient data. This includes comparing the volume of the fibrotic region between the simulation and the ground truth fibrosis data, as obtained from manual segmentations by a medical professional. Furthermore, the spatial agreement between the simulated and actual fibrotic region is determined through the use of two image similarity indices, DICE and RAND, which act complementarily to provide a comprehensive assessment of the model’s performance. Detailed explanation of both similarity indices is provided in the electronic supplementary material, §3.3, while quantitative comparison data for each patient case is listed in table 1.

**Table 1.** *In silico*-predicted volumes of the fibrotic region within the lung at two time points (each corresponding to the respective follow-up scans). For each follow-up, the columns list the volume of the fibrotic region, the deviation of the *in silico* fibrotic volume to the ground truth one and the values of the similarity indices.

patient ID	lung vol. (cm^3^)	follow-up #1				follow-up #2			
		vol. (cm^3^)	dev. (%)	DICE	RAND	vol. (cm^3^)	dev. (%)	DICE	RAND
02	1.38	46.6	0.19	0.257	0.932	128.4	0.13	0.306	0.847
03	1.88	51.3	0.01	0.250	0.943	98.0	−0.20	0.138	0.893
05	1.76	0.2	—	—	—	383.0	−0.02	0.501	0.762
06	2.42	115.8	0.20	0.213	0.904	464.7	−0.10	0.555	0.810
07	1.84	117.1	0.19	0.218	0.883	142.4	−0.12	0.370	0.895
08	1.79	46.8	0.18	0.173	0.941	115.0	0.08	0.373	0.904
09	1.75	0	—	—	—	86.4	0.15	0.231	0.906
10	1.40	13.7	−0.17	0.108	0.974	31.6	−0.05	0.010	0.938
11	1.58	44.7	−0.90	0.039	0.930	242.9	0.14	0.301	0.779
12	2.50	15.8	0.34	0.062	0.982	19.1	0.15	0.096	0.982
13	1.09	0	—	—	—	56.0	−0.01	0.329	0.913
14	1.99	112.2	0.21	0.206	0.889	158.1	−0.07	0.312	0.868

**Figure 4.**
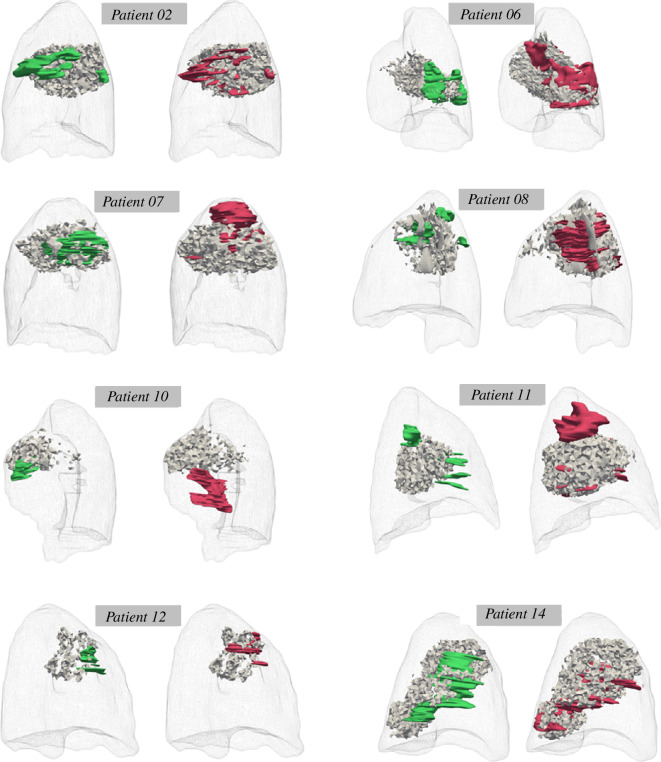
Comparison of the simulated fibrosis (grey-coloured regions) against the segmented contours that were delineated from clinical imaging data (green-coloured and red-coloured contours). The results shown to the left of each case depict the first follow-up while the second follow-up comparison is on the right, respectively.

Spatial comparison between the ground truth and the *in silico*-predicted region of lung fibrosis is performed through the DICE and RAND image similarity indices. By definition, the indices range from 0 (no overlap) to 1 (complete overlap). The spatial comparison of the simulated and the ground truth fibrotic regions results in a range of DICE index values between 0.1 and 0.56 for the majority of patients, which indicates varying degrees of overlap between the clinical measures and the model output of lung tissue scarring. The RAND index values are consistently larger than 0.8 which demonstrates high level of agreement. Visual comparison between the simulated fibrotic regions and the segmentations for six representative cases (Patients 02, 06, 07, 08, 10, 11, 12 and 14) is provided in figure 4. The figure 3*d* overlays of the simulated fibrotic regions (depicted in light grey) and the fibrotic regions identified by the medical expert (marked in green for the first follow-up and red for the second). As can be seen in the majority of cases, there is a good agreement in terms of general location and extent of the fibrotic region. However, details in the shape of the fibrotic regions differ between the simulated and actual results. In particular, the simulated regions often appear more uniform or smoother compared with the more irregular shapes of the actual fibrosis.

It is important to note that the spatial comparison has revealed some cases with very little overlap and a low DICE index. In particular, the second follow-up of Patient 10 gives a simulated fibrosis region that does not overlap with the fibrosis segmentation, thus, giving an extremely low DICE index result 0.003. This is mainly a case-specific issue because this patient has been observed to develop pulmonary fibrosis in entirely different parts of the irradiated lung between the first and second follow-up CT scan. As RIPF is a progressive disease, the fibrotic region in the second follow-up is expected to overlap significantly with the first one, which is not true in this case. This indicates difficulties in accurately capturing the tissue damage for Patient 10, which influences the agreement with the simulated results, as the model is formulated to follow the progressive nature of fibrosis. Furthermore, cases with a very small extent of RIPF (e.g. Patients 10 and 12) are inherently more prone to low DICE index because they can have very little overlap even though the *in silico* model predicts fibrosis in the same general area as indicated in the ground truth data. This is evident for example in Patient 12, who has a dice index of less than 0.1 even though the simulated fibrosis develops in the same region as shown in figure 4 (bottom left). Finally, there are some cases which show parts of the fibrotic regions that are not entirely captured by the simulation (e.g. at the apex of the lung in Patients 07 and 11). This suggests the presence of additional effects influencing fibrosis development that are not accounted for in the model or some patient-specific characteristics that the model does not include.

Overall, the results demonstrate a high degree of qualitative agreement between the simulated and actual fibrosis volumes for both follow-ups for all patient cases. However, the spatial overlap metrics (DICE and RAND index) exhibit some variation, indicating that the model’s accuracy in predicting the precise location of fibrosis is less consistent. This suggests that while the model is able to capture the overall volume of fibrosis, further refinements are necessary to improve its ability to reproduce the exact spatial distribution of fibrotic tissue.

### 3.3. Hounsfield units dynamics reflects on lung tissue remodelling

The third state variable of the *in silico* model, ϵ, represents the radiodensity of lung tissue and is based on the CT data (expressed in HU). Its rate of change depends on the rate of change of tumour and fibroblast cells. As indicated in equation (2.3), the cancer and fibroblast rates are weighted by coefficients $\phi_{c}$ and $\phi_{f}$, respectively. These coefficients have been carefully selected to ensure that they produce physiologically relevant HU values throughout the process of tumour regression and fibroblasts proliferation and migration. Specifically, the selected values for the two coefficients lead to a reduction of the HU of the tumour area to levels typical of healthy lung tissue (approximately −900 to −700 HU) as well as produce typical values for fibrotic tissue (approximately 0–100 HU) as the fibroblast population increases. Importantly, the coefficients $\phi_{c}$ and $\phi_{f}$ are uniform across all patients, irrespective of other model parameters, and have values $2.5\;\times \;10^{3}\;$ and $15\;\times \;10^{3}\;HU$, respectively.

**Figure 5.**
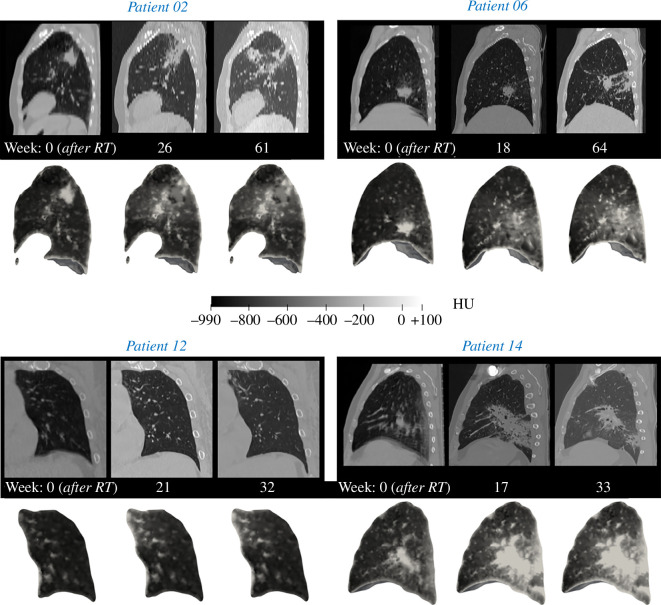
Comparison of the ground truth CT images from the baseline and follow-up scans (top row) against the simulated radiodensity contours on the lung coloured in HU scale (bottom row) for four representative patient cases. For ease of visual comparison, the greyscale range is kept uniform for all images as shown in the centre of the figure.

Figure 5 depicts the results for four indicative cases (Patients 02, 06, 12 and 14), where the simulated HU data (i.e. ϵ) are examined against the ground truth CT imaging data, starting from the baseline to the two follow-up scans. The comparison demonstrates a very good qualitative agreement between the simulated and actual scans for all cases, which vary significantly in terms of fibrosis extent. Patients 02 and 06 show extensive fibrosis, which is represented in the HU field of the simulations at largely the same regions. Patient 12 has minimal fibrosis development, which can be seen only on the top left of the coronal slice of the second follow-up. The agreement consistency across different patients underscores the robustness of the *in silico* model to realistically reflect tissue changes over time. By accurately replicating radiodensity values seen in the CT images, the model can facilitate a direct understanding of the progression and spatial distribution of fibrosis, potentially benefiting treatment planning and monitoring of the disease.

## 4. Conclusions

In this contribution, we have developed an innovative computational procedure to simulate the occurrence and progression of pulmonary fibrosis in lung cancer patients who have undergone RT to the chest. The *in silico* model is personalized, utilizing the patient’s lung geometry and incorporates structural information (i.e. tumour size and location, lung tissue radiodensity) from CT scans as well as the amount of radiation dosage delivered from external beam RT. The model is capable of simulating lung tumour regression, fibrosis development and corresponding changes to the lung tissue, which is represented by the HU scale, used in CT images.

The model was calibrated using data from 12 lung cancer patients, which included baseline scans prior to RT, the radiation dosage plans and two follow-up scans that show the respective fibrotic regions in the organ. The analysis of data from the patient cohort has shown that the extent of fibrosis at the first follow-up correlates well with the total radiation received by each patient (r=0.63). However, the second follow-up, which is more representative of the final stage of RIPF, shows the strongest correlation with the volume of tissue receiving one of the highest doses in the range of 50–60 Gy (r=0.60). This is in agreement with the general consensus that the highest doses of radiation are responsible for lung injury and subsequent fibrosis development [41]. The *in silico* model shows excellent agreement with respect to the extent of fibrosis at both follow-ups, with less than 1% deviation from our clinical data, used here as ground truth evidence. The agreement in terms of the location and shape of the predicted fibrotic region varied, with DICE indices ranging from 0.3 to 0.6 in most cases. In two cases (Patients 10 and 12), the model produced poor agreement results in terms of the DICE coefficient—this can be partly attributed to the fact that these cases had small regions of fibrosis, making it more challenging to achieve a good overlap with the simulations. Additionally, inherent uncertainties in the identification of fibrosis in patient scans contribute to model predictions discrepancies. This is evidenced by the fact that two patient cases (i.e. Patient 03 and Patient 10) had completely distinct regions of scarred tissue between the first and second follow-up. In terms of the RAND coefficient, the second metric used for the validation, which considers the image similarity within the whole lung, the agreement is consistently high for all cases (above 0.8 for the large majority).

The distribution of model parameters across patients, evaluated through a calibration process, revealed important characteristics of the model and its relation to the imaging data. The mathematical terms describing fibrosis spread (diffusion and haptotaxis transport parameters in equation (2.2)) have small variance between patients with the coefficients of variation calculated at 0.020 and 0.025, respectively. However, the model parameters of the mathematical terms describing fibrosis growth (i.e. the recruitment and proliferation parameters in equation (2.2)) have higher variation with coefficients of variation ranging from 0.3 to 0.5. This illustrates their dominance in determining the extent of fibrosis, which itself varies significantly among patients—the coefficient of variation is calculated at 0.9 approximately across both follow-ups. In terms of the relation of the model parameters to the extent of fibrosis, parameter $\lambda_{f}$ shows positive correlation with the volume of fibrosis across both follow-ups (correlation index of 0.61), which illustrates that patients with higher fibrosis development will have higher production coefficient values. However, the parameter for fibroblasts’ recruitment, $\kappa_{f}$, and death, $\theta_{f}$, did not show correlation with the extent of the fibrosis, which demonstrates the level of model complexity, as these rates are not merely proportional to the magnitude that pulmonary fibrosis manifests in each lung cancer patient.

Although the *in silico* model demonstrates promising results in simulating RIPF, we recognize several limitations that point to important directions for future research and model refinement. The tumour evolution parameters have not been calibrated to this patient cohort and were obtained from literature, due to lack of data on tumour regression. Thus, the simulated tumour evolution is a typical representation of tumour regression, which allows for capturing the interaction of tumour cells with other variables in the model while offering the opportunity to incorporate patient-calibrated parameters when available. Furthermore, chemotherapy treatment is not considered in this model of RIPF. Since all lung cancer patients received adjuvant chemo, its effects were assumed to be uniform across the patient cohort. Despite this, individualized treatment conditions, minor differences in chemotherapy and particularly the use of immunotherapy may affect the progression of fibrosis [3]—this can be investigated in future extensions of the model, yet new pertinent imaging data from a prospective clinical study is required. Additionally, another improvement of the *in silico* model is the inclusion of lung biomechanics that will enable the study of lung deformations and changes to lung capacity due to tissue stiffening caused by RIPF. However, identifying the appropriate modality of data to inform and personalize the model in this direction is elusive.

In conclusion, the simulation findings of this study are promising, demonstrating that a relatively simple *in silico* model can effectively represent the evolution of fibrosis in lung cancer patients post-RT. The ability of the model to closely match the extent and location of the fibrotic regions observed in patient data underscores its potential for clinical translation. Future studies will leverage this model to extract deeper insights into the mechanisms of fibrosis development and assess its predictive capability on a larger lung cancer patient cohort. The parameter set created in this study can be statistically analysed to extract representative parameters for the general population, providing a foundation for predicting fibrosis in new patients. This approach can be further refined by incorporating patient-specific information beyond the RT plan, such as age, underlying conditions and medication history, to tailor the model more closely to individual patients. As more patient data become available, techniques like Bayesian inference or machine learning models can be employed to predict plausible parameter sets for new patients based on the data from the calibrated patients. This demonstrates significant promise towards improving cancer patient care and outcomes against RIPF.

## Data Availability

The code and data from this study are available at the Zenodo repository [42]. The code is also available at the GitHub repository [43]. Supplementary material is available online [44].
